# Glycogen Storage Disease Type IX due to a Novel Mutation in PHKA2 Gene

**DOI:** 10.1155/2020/8836534

**Published:** 2020-09-18

**Authors:** Hamza Hassan Khan, Lauren Parr, Allison Jay, Saleem Raza, Hernando Lyons, Sanjay Kumar

**Affiliations:** ^1^Department of Pediatrics, Ascension St. John Children's Hospital, Detroit, MI, USA; ^2^Department of Medical Genetics, Ascension St. John Children's Hospital, Detroit, MI, USA; ^3^Department of Pediatrics, Ascension St. John Children's Hospital, Wayne State University, School of Medicine, Detroit, MI, USA; ^4^Department of Pediatric Gastroenterology, Ascension St. John Children's Hospital, Wayne State University, School of Medicine, Detroit, MI, USA

## Abstract

We report a case of a 17-month-old male with a history of developmental delay with poor muscle control, hepatomegaly, and transaminitis. Ultrasound of abdomen revealed hepatomegaly with a liver span of 13 cm, homogeneous parenchyma, and normal spleen size. Liver and muscle biopsies were obtained: the liver biopsy revealed distended hepatocytes with excessive glycogen accumulation and fine septate fibrosis. Biopsy of the right vastus lateralis muscle showed focal swollen glycogen containing mitochondria. For the developmental delay, a chromosomal microrarray was ordered. The chromosomal microarray revealed the patient to have 1q21 duplication syndrome and 16p11.2 deletion syndrome. Given the liver and muscle biopsy findings, a glycogen storage disease panel was sent which identified the patient to be hemizygous for a variant of uncertain significance denoted as p.Gly 131Val, c.392G > T in the PHKA2 gene. PKHA2 gene encodes the alpha subunit of hepatic phosphorylase kinase. This change in the PHKA2 gene was in a highly conserved region and had been reported in another patient with decreased enzymatic activity of the phosphorylase kinase and who had symptoms of GSD IX. Based on this, the patient was started on treatment for GSD IX, and his family met with a dietician.

## 1. Introduction

Glycogen storage diseases are inborn errors of carbohydrate metabolism that affect the storage, release, or consumption of glycogen. Glycogen is a branched polymer of glucose that serves as a form of energy storage for the body to be later converted into glucose. There are over 13 different types of glycogen storage diseases identified based upon the enzyme that is dysfunctional or absent. The incidence of glycogen storage diseases is estimated to be 1 per 20,000–43,000 births [1].

Amongst different types of glycogen storage disease, type IX accounts for 25% of cases [2]. In particular, the subtype IXa, caused by a mutation in the *PHKA2* gene, accounts for approximately 75% of cases in GSD IX [1]. Due to the broad phenotypic traits and genetic heterogeneity, it remains challenging to make the diagnosis of glycogen storage disease type IX.

## 2. Case Presentation

A 17-month-old African-American male was referred to the pediatric gastroenterology clinic at a community hospital with a history of hepatomegaly, developmental delay, poor muscle control, and transaminitis. The patient was born to a 23-year-old gravida 2, para 2 mother, by an uncomplicated spontaneous vaginal delivery at 39 weeks of gestation. His newborn course was unremarkable. Newborn screening through the Michigan Department of Health and Human Services was found to be within normal limits in all categories. At three weeks of age, he was diagnosed with pyloric stenosis and underwent a successful pyloromyotomy. The family lost follow-up with his pediatrician until fifteen months of age due to poor socioeconomic situation—family was homeless and was living in a shelter. At that time, the patient was found to have significant developmental delay; his mother reported that the patient started sitting at the age of 9 months and crawling when he was 1 years old. The patient was primarily nonverbal. His mother denied any history of jaundice, excessive bruising, itching, dark urine, pale stool, abdominal pain, vomiting, or poor appetite. There was no family history of liver diseases including hepatitis and other infectious etiologies. On laboratory workup, he was identified to have low hemoglobin (Hb) of 7.9 and hence was started on iron supplementation with the plan of follow-up with the primary care pediatrician (PCP) in 2 months.

On follow-up visit at 17 months of age, the patient was noted to have poor muscle strength, could only stand with support, was still nonverbal, and his abdomen was noted to be distended with significant hepatomegaly which prompted the PCP to initiate diagnostic workup and pediatric gastroenterologist referral.

Diagnostic workup was performed which revealed Hb: 9 gm/dL, reticulocyte count: 2.5%, erythrocyte sedimentation rate (ESR): 51 mm/hr, prothrombin time (PT): 13.5 seconds, international normalized ratio (INR): 1.06, glucose of 40 mg/dL with normal subsequent readings, bicarbonate (HCO_3_^−^): 20 mmol/L, anion GAP: 19 mmol/L, serum glutamic oxaloacetic transaminase (SGOT): 470 unit/L, serum glutamic pyruvic transaminase (SGPT): 531 unit/L, gamma-glutamyl (GGT): 229 unit/L, albumin: 4.3 gm/dL, creatinine kinase (CK): normal, lactate dehydrogenase (LDH): 1115 unit/L, aldolase 27.2 unit/L, uric acid: normal, lactic acid: 5.7 mmol/L, pyruvate: 0.184 mmol/L, and cholesterol: 353 mg/dL (HDL: 15 mg/dL, LDL: 237 mg/dL, and triglycerides: 643 mg/dL). His hepatitis panel and antinuclear antibody (ANA) was negative, and antismooth muscle antibody (ASMA) was 1 : 40.

**Figure 1:**
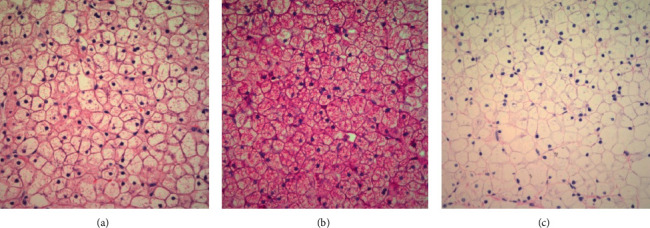
(a) Hemotoxylin and eosin (H&E) stain showing enlarged and distended hepatocytes with clear cytoplasm. (b) Periodic acid-Schiff (PAS) stain showing increased glycogen content in the cytoplasm of hepatocytes. (c) Periodic acid-Schiff-diastase (PAS-D) stain showing the removal of glycogen from hepatocytes with the diastase staining.

Ultrasound of abdomen was performed which revealed hepatomegaly with a liver span of 13 cm, homogeneous parenchyma, and normal spleen size. Ophthalmology examination was normal without appreciation of cherry red spot on the macula. Liver and muscle biopsies were performed; muscles biopsy was performed as he had poor muscle strength and we wanted to assess if there are glycogen deposits in the muscle. The liver biopsy revealed distended hepatocytes with excessive glycogen accumulation and fine septate fibrosis, and the biopsy of the right vastus lateralis muscle revealed uneven distribution of mitochondria in muscle fibers as well as focal swollen glycogen containing mitochondria (Figure 1).

Liver biopsy tissue specimen was sent for glycogenosis liver assay using a spectrophotometer. The liver glycogen content was found to be elevated at 10.1 *μ*molgluc/mg tissue (reference range of 1.6–5.1 *μ*molgluc/mg tissue). Glucose-6-phosphatase, phosphorylase, and debrancher enzyme were all found to be within the normal limits.

The patient was referred to a geneticist at an academic institution, and a glycogen storage disease panel was sent to evaluate his liver disease. This found a change c.392G > T in the PHKA2 gene, classified as a variant of uncertain significance. Given this change was in a highly conserved region and had been reported in another individual with similar symptoms, the treating geneticist decided to follow-up the patient according to the American College of Medical Genetics Guidelines for GSD IX. The patient also had a chromosomal microarray ordered for his developmental delay and was found to have two copy number changes related to delays, namely, 16p11.2 deletion and 1q21 duplication syndrome.

The patient was evaluated by a dietician who placed him on a high protein and high complex carbohydrate diet. He was recommended to consume uncooked cornstarch mixed in his evening cup of milk. His mother was provided with a glucometer and was instructed to check glucose levels daily. She was also instructed to prevent prolonged fasting and to wake him up around 3 am for a snack to avoid overnight fasting.

Pediatric cardiology was also consulted due to concerns of cardiac involvement with glycogen storage diseases. The patient was found to have mild left ventricular hypertrophy (LVH) on echocardiogram but otherwise preserved cardiac function. The mother was instructed to follow-up in 6 months to reassess for worsening LVH or sooner if new cardiac symptoms arose.

In addition, due to the fact that GSD IX, caused by the PHKA2 gene, is inherited in the X-linked recessive pattern, the mother was counselled and encouraged to undergo genetic testing to see if she poses a risk for her other children. If the mother is found to be positive for the variant gene, it was suggested that other family members would need to undergo genetic testing as well.

## 3. Discussion

GSD type IX is caused by a deficiency in the phosphorylase kinase enzyme. This enzyme plays a regulatory role in the breakdown of glycogen into glucose-1-phosphate and consists of 4 different subunits. *α*, *β*, *γ*, and *δ* subunits of PK are encoded by four different genes: PHKA1, PHKA2, PHKB, and PHKG2, respectively. The PHKA1 gene encodes the muscle form of phosphorylase kinase, and the PHKA2 gene encodes *α* subunit for the hepatic phosphorylase kinase [3].

There are over 80 mutations reported by the Human Gene Mutation Database of the PHKA2 gene that is associated with GSD Type IXa; these mutations include small deletions, gross deletions variants, small insertions, missense, and splicing [4]. Clinical manifestations typically become apparent within a few months of age and may present with a number of findings such as a protuberant abdomen secondary to hepatomegaly, elevated transaminases, hypertriglyceridemia, hypercholesterolemia, ketosis with or without hypoglycemia, growth retardation, and delay of motor development. Some cases also report a characteristic rounded facies that tends to resolve with age [5]. A mutation in the PHKG2 gene affecting the *γ* subunit is associated with the most severe phenotype as it carries a high prevalence of liver fibrosis which can develop in childhood [6].

GSD type IX has been found to be prevalent amongst the Chinese population [7–9]. A former literature review compared 21 cases of GSD IX amongst Chinese individuals. These cases consisted of males ranging from 3 months to 10 years. Out of those 21 cases, 19 were identified to have mutations of the PHKA2 gene [7–9]. Amongst those cases, 95% had transaminitis, 91% had hepatomegaly, 43% had growth retardation, 43% had hyperlipidemia, and 38% were found to have fasting hypoglycemia [8]. Delayed motor development was noted to be rare, as only 2 cases have been identified with delayed motor development [8, 9].

The physical manifestations of GSD IX often overlap with the other types of glycogen storage diseases and genetic syndromes ultimately making the diagnosis difficult and the initiation of treatment delayed. The average time of diagnosis of GSD IX has been found to be approximately 6 years reflecting the existing challenges in the diagnosis [8]. In addition to the classic findings of GSD IX such as hepatomegaly, hyperlipidemia, and transaminitis, our patient was also found to have significant developmental delay which is likely attributable to the 1q21 duplication syndrome and 16p11.2 deletion syndrome discovered on genetic testing. 1q21 duplication syndrome has been reported to be associated with abnormal head size, developmental delay, autism or autistic behaviors, as well as a broad range of clinical anomalies such as congenital heart defects, esotropia, and lower limb hypertonicity [10]. 16p11.2 deletion syndrome has been reported to have a high frequency of global developmental delay, particularly autism, as well as associated learning and language difficulties [11].

There is a specific growth pattern that is found to be associated in males with X-linked GSD IX. Typically, these affected individuals are found to have a normal height at birth, followed by substantial growth retardation between the ages of 2 and 10 years of age, significantly delayed puberty, and then go on to attain normal adult parameters [12]. Our patient's height trended downwards since his evaluation at 15 months of age; he was at 37 percentile at 15 months, 10 percentile on 17 months visit, and only 5 percentile upon his last visit at 30 months of age. Often no intervention is needed for growth delay; however, frequent monitoring will be needed in this patient in order to prevent long-term complications. Adverse effects including pubertal delay can in turn cause reduced bone density and increased risk of bone fractures in adulthood [12].

Hepatomegaly and elevated transaminases have been found to improve without intervention over the first two decades of life. However, frequent monitoring of these markers and liver ultrasound should be performed every 1-2 years in order to monitor complications such as liver cirrhosis. Studies have shown that aggressive structured dietary treatment with uncooked cornstarch and high protein intake resulted in increased improvement of hepatomegaly, energy, growth velocity, and a decrease in sonographic features of fibrosis when present [4, 13].

In a case series, interventricular septal hypertrophy was discovered in a patient with GSD type IX due to a mutation in the PHKB gene [13]. Asymptomatic left ventricular hypertrophy was only noted to be present in a patient with GSD VI [13]. Our patient was found to have asymptomatic LVH with GSD type IXa involving the PHKA2 gene. There are limited data involving cardiac manifestations in patients with GSD IX, and further studies are needed to analyze the utility of cardiac testing. However, it is recommended that patients diagnosed with GSD should undergo echocardiogram every 12–24 months after 5 years of age.

Conclusively, our case report furthers knowledge about PHKA2 gene mutation which can lead to glycogen storage disease type IX and its different phenotypic characteristics. There still remain many challenges in the diagnosis and caring for patients diagnosed with a glycogen storage disease. Further studies need to be done in order to close the current knowledge gaps and improve treatment recommendations and guidelines for managing patients with GSD. Furthermore, patients with glycogen storage diseases may have other concurrent genetic findings to explain developmental delays outside of what is expected for the specific disorder.
